# Antimicrobial and Osseointegration Properties of Nanostructured Titanium Orthopaedic Implants

**DOI:** 10.3390/ma10111302

**Published:** 2017-11-13

**Authors:** Marcus Jäger, Herbert P. Jennissen, Florian Dittrich, Alfons Fischer, Hedda Luise Köhling

**Affiliations:** 1 Department of Orthopaedics and Trauma Surgery, University of Duisburg-Essen, Hufelandstrasse 55, D-45147 Essen, Germany; hp.jennissen@uni-due.de (H.P.J.); florian.dittrich@uk-essen.de (F.D.); 2 Institute of Physiological Chemistry, University Hospital, University of Duisburg Essen, Hufelandstrasse 55, D-45147 Essen, Germany; 3 Institute of Metal Engineering, University of Duisburg-Essen, Campus Duisburg, Lotharstraße 1, D-47057 Duisburg, Germany; alfons.fischer@uni-due.de; 4 Institute of Medical Microbiology, University of Duisburg-Essen, Virchowstr. 179, D-45147 Essen, Germany; Hedda-Luise.Koehling@uk-essen.de

**Keywords:** nanostructure, implants, bone, antimicrobial, titanium, protein layer

## Abstract

The surface design of titanium implants influences not only the local biological reactions but also affects at least the clinical result in orthopaedic application. During the last decades, strong efforts have been made to improve osteointegration and prevent bacterial adhesion to these surfaces. Following the rule of “smaller, faster, cheaper”, nanotechnology has encountered clinical application. It is evident that the hierarchical implant surface micro- and nanotopography orchestrate the biological cascades of early peri-implant endosseous healing or implant loosening. This review of the literature gives a brief overview of nanostructured titanium-base biomaterials designed to improve osteointegration and prevent from bacterial infection.

## 1. Introduction

Titanium (Ti) is a bioinert metal which is frequently applied in orthopaedic surgery for decades. In contrast to other metals such as cobalt-chrome-molybdenum (CoCrMo) or surgical steel (CrNiMo), it has almost no allergenic or immunogenic potential in vivo, showed excellent corrosion resistance and promotes osseointegration when applied to and fixed with osseous tissue. In addition, the low Young’s modulus of titanium and its alloys has biomechanical advantages since it results in smaller stress shielding compared to other biometals (Table 1). In contrast to pure commercial titanium (cp-Ti), the alloy Ti-6Al-4V is commonly used in orthopaedic implants. One prominent candidate is total joints. Here, it was shown, that the macro- and microsurface structure influences the behaviour of protein adsorption as well as local cell adherence, proliferation and differentiation. Aiming for a high initial stability at the bone–implant interface, different manufacturers have modified and optimized surface structures. Typical examples are:

- the increase of the surface area by sandblasting;
- the surface coating with hydroxyapatite (HA) deposit; and
- the increase of macroporosity such as “metal spongiosa”.

To date, the variety of surface modifications for titanium is extensive including mechanical (gritt blasting and attrition), chemical (acid etching and electrochemical depositions) and physical techniques (plasma-spraying) or a combination of these [1] (Table 2).

In contrast to its excellent osteoconductive properties, the low hardness of titanium exhibits a low wear resistance and limits its application for articulating parts of artificial joints. Another major disadvantage of most Ti surface designs is that they do only promote initial cell adherence followed by osseointegration but also promote bacterial adherence. The winner in the “race for the surface” (cell versus bacteria) decides if biomineralization leads to a solid anchoring of a total joint or bacterial growth and biofilm deposit leads to the clinical incidence of a periprosthetic infection (PI). The treatment of PI is demanding for both the orthopaedic surgeon and the patient. Even in uncomplicated cases, it is associated with two or more operations and requires mid-term immobilization of the patient. Moreover, PI results in relevant bone loss and soft tissue defects leading to reduced muscular function and may result in poor implant survivorship and disability of the patient. Especially multiresistant bacteria are problematic since their escape from eradication by antibiotics. Therefore, strong efforts have been made to develop innovative implant surface structures, which promote osseointegration as well as prevent PI. Since nanopatterning with dimensions smaller than 100 nm has entered orthopaedic research, one alternative is to design surface structures at the nanoscale, which are promising to reach these aims. This review of the literature summarizes recent data of nanostructured titanium implants with emphasis on orthopaedics.

## 2. Bone Cells and Their Interactions

To predict the response of an osseous microenvironment to nanostructured titanium biomaterial in vivo, it is essential to get a brief insight into the complexity of the osseous cellular interactions in general and in the cell–surface interface in detail. Bones have multiple and various functions. The vital bone structure, by definition a rigid organ, is part of the hematopoietic system, stores minerals, supports and protects vulnerable organs, provides structure for the human skeleton and enables mobility. Depending on the constantly changing biomechanical requirements, bone adapts its structure. This is physiologically conditioned by a continuous balance of bone formation and degradation. This bone remodelling process is influenced by systemic (e.g., calcitonin and oestrogens) as well as local (e.g., growth factors and cytokines) stimuli [25]. Moreover, osteocytes act as mechanosensors and orchestrators of this homeostasis. Osteoblasts are able to produce osteoid (a collagen I rich matrix) and additional capable of osteoid calcification (hydroxyapatite). An adequate stimulus derives the active osteoblast, characterized by a large amount of endoplasmatic reticulum as well as a large Golgi apparatus, from its mesenchymal progenitor cell which is mainly localized in the bone marrow [26].

In contrast, osteoclasts maintain the homeostasis between bone formation and resorption as antagonists of the osteoblasts. They are characterized by multiple nuclei and are derived from the hematopoietic cell line. The homeostasis of bone formation and degradation is complex and predominantly regulated by the receptor activator of nuclear factor κβ (RANK) and its associated ligand. Receptor activator of nuclear factor κβ ligand (RANKL) does not only activate downstream pathways of osteoclastogenesis, but also modulates the homoeostasis of physiological as well as pathological osseous processes by linking to other signal paths [27]. During differentiation, the osteoblast progenitor cells express stimuli of osteoclastogenesis factors like macrophage colony stimulating factor (M-CSF) and RANKL. In contrast, osteoprotegerin can antagonize RANKL by preventing binding to the receptor RANK.

In addition to the primarily bone forming cells, the osseous milieu contains its genuine and high specialized immune system. Nevertheless, the transition between immune and bone forming cells is fluent. The formation of osteolysis is commonly associated with the activity of osteoclasts as primarily bone-resorbing cells. In contrast, some studies showed that macrophages or multinuclear giant cells, which are ontogenetically closely linked to osteoclasts, are also capable of low-grade bone resorption [28]. The macrophages’ dual function as both immune and bone degrading cells is just an exemplary demonstration of the complexity and tight interaction of the osseous milieu.

## 3. Titanium Surface Properties and Osteointegration

It is evident that the osseous response depends not only on the biochemical composition of a biomaterial but also on the biomechanical characteristics of the implants surface. Topographical factors such as size, surface texture, macro- and microstructure, and shape of the implant play a crucial role in the cellular reaction [29]. The biomaterial effects in terms of bioinertness, bioactivity, and biocompatibility are important in implantology as well as biomechanical characteristics. Biocompatibility means the ability of a material to induce an adequate reaction in the host (human, animal, organ, and cell) in a specific situation [30].

Titanium (Ti) as a feedstock and Ti-based alloys have been widely used as implant biomaterials in orthopaedic surgery in the last sixty years because of their genuine biomechanical and biocompatible characteristics. In comparison with CrNiMo stainless steel, Ti has a 50% greater strength to weight ratio and, in contrast to CoCrMo, it has almost no allergenic or immunogenic potential in vivo. Moreover, the rapidly forming durable dielectric titanium dioxide layer on the implant surface induces cell integration, enabling a much stronger contact between Ti-based implants and bone tissue after implantation compared to steel [31]. The cause of this continuous protective and thermodynamically stable oxide layer is a very high affinity of titanium for oxygen (so called “passive film”). Thus, disruptions or damages are repaired immediately when bioliquid surrounds the metal [32]. The detailed composition of this passive film in vivo is mostly based on TiO_2_ while any small content of Ti_2_O_3_ and TiO depends on the local micro-environment [33,34].

The thickness and quality of the passive condition in titanium can be controlled by thermal treatment. High temperatures (up to 800 °C) and longer thermal oxidation times cause oxide de-bonding, whereas oxide layers obtained at lower temperatures and shorter times are also not sufficiently thick.

In aqueous solutions such as body fluids, the passive film under mechanical stress is not a static condition but might be characterized by continuous depassivation and repassivation. Here, alloying elements and impurities such as molybdenum (Mo), niobium (Nb), vanadium (V) or chromium (Cr) are probably not involved to significant content. Nevertheless, dissolution of alloying elements during time is possible, as well as incorporation of different elements from surrounding solutions into the passive film. These effects might play a role in orthopaedic implants since “repassivation of titanium” at the osseous implantation site led to the adsorption of calcium and phosphate ions into the film, whereas at the outermost surface calcium phosphate and calcium titanium phosphate were formed [35,36,37]. In contrast to cp-Ti, the alloy Ti-6Al-4V is commonly used in orthopaedic implants for its better mechanical properties [38].

It is generally accepted that the biocompatibility of titanium can be improved by the coating with bioactive titanium dioxide and by the presence of structures at micro- and nanoscales [39]. Especially plasma spraying, chemical vapour deposition, atomic layer deposition anodic oxidation, sol-gel and other methods have been established for nano-designs [40]. However, osteointegration is in first turn conditioned by osteoconduction (adaptive bone tissue growth/ingrowth). Therefore, not only nano-scale structures but also surface topography at the macro- and microscale are relevant for the success of an orthopaedic implant.

### 3.1. Local Biomolecular Reactions to Titanium Surfaces

The detailed cellular mechanisms on the bone–implant interface during osteogenesis are still largely unknown. The first response after application of titanium to an osseous environment is wetting followed by rapid protein adsorption to its surface [41].

The capacity of an implant surface to adsorb small proteins depends on its physicochemical characteristics such as hydrophobicity or surface energy and the local milieu (pH, temperature, concentrations of ions, composition and functional groups of proteins, strength of solution) [42].

The first proteins that rapidly adsorb at the solid–liquid interface in vivo usually derive from blood. These interact with the surface structure and undergo conformational changes, such as fibrinogen adopting a fibrin-like structure, depending on the surface type and the exposure time. Here, it is evident that the composition of the adsorbed protein layer varies and proteins with stronger adsorption are favoured.

During the next minutes and hours this initial phase is replaced by a formation of a resident protein layer, which influences the interaction of platelets, activation of intrinsic coagulation, adhesion as well as aggregation of platelets and activation of the complement system [43]. Especially the Arg-Gly-Asp (RGD) sequences of the proteins fibronectin and vitronectin play a crucial role as chemotactic or adhesive stimuli in biomineralization and matrix maturation [44,45]. In addition, the implant surface has a strong impact on the platelet activation level. Here, some blood coagulation factors are triggering formation of thrombin followed by converting fibrinogen into insoluble fibrin. Fibrin is retained by titanium surface structures as a transient matrix. The fibrin retention forces are strongly influenced by the local topography of the implant surface. Therefore, fibrin is not only involved in initial cellular adherence but also concomitant with cell traction.

Once the biomaterial is coated with a transitory protein layer, different cell types attach rapidly to the protein-conditioned surface [46]. This initial attachment follows an adhesion phase, dominated by interactions of the extracellular matrix (ECM) proteins and adjacent cells. Integrins play a crucial role as “molecular bridges” between adherence proteins and adsorbed ECM proteins of the biomaterials surface and are able to transduce information about biomechanical load from ECM proteins to the peripheral cytoskeleton. This interaction leads to the activation of gene promotors via nuclear matrix architectural transcription factors (NMATF) and finally to the release of chemotactical signals [47]. Osteogenic cell migration, proliferation and differentiation follow in the next phase. One can highlight that bone cells align along defined morphologies but the underlying interactions with the nanotopography are complex and not fully understood in detail. Mainly responsible for the final osseointegration of titanium biomaterial and bone formation are osteoblasts. However, under pathological conditions, protein adsorption can be reversible, especially if the local pH value decreases, as in inflammation [48,49,50].

**Figure 1:**
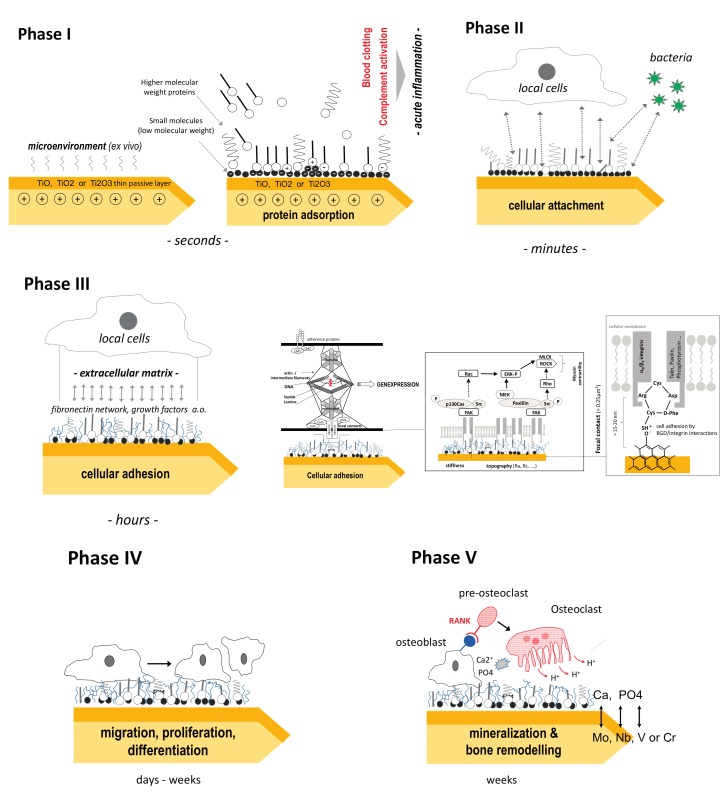
Local reactions onto titanium surfaces after in vivo application.

Figure 1 gives more detailed information on the different interactions between Ti surfaces and its in vivo microenvironment which can divided into the following five phases:

**Phase I**: Adsorption of low and higher molecular molecules. The positive charge of titanium is responsible for an initial binding of negative charged small molecules followed by negatively loaded functionalized groups of high molecular weight molecules. In this dynamic stage, local proteins (e.g., fibronectin, albumin, fibrinogen, IgG, complement C3 from blood and bone marrow, and vitronectin) are mainly involved, whereas lipids play a subordinate role. The type of binding is non-covalent (hydrophobic interactions, electrostatic forces, hydrogen bonding, and Van der Waals forces). With time, molecules with greater affinity for the surface but slower diffusion rates replace the smaller molecules (“*Vroman effect*”) [42]. This protein adsorption is associated with conformational change of the tertiary protein structures and changes the initial surface characteristics of the titanium implant [51]. This affects parameters such as wettability, surface energy, and hydrophilic qualities. The result in vivo is a more or less (polar) functionalized interface, which is ready now for cellular attachment (“conditioning film”). Fibronectin and collagen promotes osteoblast adhesion and osteointegration whereas albumin inhibits cellular attachment and spreading [52]. Typical example of the influence of titanium surfaces on protein adsorption are increased albumin adsorption rates in laser-engineered porous titanium [53] or higher fibronectin adsorption and lower albumin adsorption rates in magnesium-incorporated anodized titanium surfaces compared to blasted surfaces [54]. However, the detailed sequence and kinetics of competitive protein adsorption in vivo remains unclear. From 218 identified human serum proteins, 30 were detected to be associated with bone metabolism. Of these, Apo E, antithrombin and protein C are found predominantly on sand-blasted or acid-etched Ti, whereas proteins of the complement system (C3) prefer adsorption onto smooth Ti surfaces [55].

**Phase II**: In this stage, it is crucial for local cells or bacteria bind to the protein film (“the race for the surface”). Most nanostructured titanium surfaces promote both cellular and bacterial attachment. At this level, a preliminary decision of the further fate of the implant is determined (PI vs. osteointegration). Recently wound healing macrophages (M2) have been implicated as crucial for initiation of osseointegration of implants.

**Phase III**: The cells attached initially are fixed by extracellular matrix anchoring proteins to the surface of the titanium implant (non-specific cellular adhesion).

Besides mesenchymal cells, neutrophils and macrophages colonize the implant, leading to the foreign body covering process and releasing cytokines and growth factors including BMP-2 to attract fibroblasts and osteoblasts. This phase is also accompanied with a quantitative and qualitative change of the protein layer between the cell and titanium surface. The interface biology of implants is complex. Especially the ubiquitous dimetric glycoprotein fibronectin (FN), with its two subunits, is involved in this process since it binds to cellular transmembrane integrins (e.g., integrin α_5_β_1_ binds the arg-gly-asp [RGD]-containing repeat of FN and acts as a primary receptor). FN also has growth factor binding regions and is able to fix growth factors for the corresponding receptors of the cellular membrane, e.g., synergistic effects on FN and bone morphogenic proteins (BMPs). Although rough titanium surfaces exhibit a better cellular attachment, some data indicate that smooth surfaces promote cellular spreading more than rough topography [56,57,58].

The initial layer of predominantly negatively charged proteins onto the titanium surface neutralizes its charge during phase I and II. Afterwards also positive loaded extra- or intracellular proteins and ions (e.g., Ca^2+^) bind to the negatively charged functional groups of the protein layer. One prominent example are the positive loaded histones from cellular fragments or from cytosol that bind to the interface of nanostructured titanium [59,60]. The epigenetic function of these proteins remains unclear.

**Phase IV**: Controlled by titanium surface structures and its interface molecules and mediated by focal cellular contacts, local growth factors and intercellular cytokine proliferation and differentiation are induced. Focal adhesions (contacts) are dynamic structures and link the cytoskeletal network to the ECM, allowing cells to respond to the external environment (feedback loop: inside-outside-inside signalling). Therefore, mechanical forces at the implant-cellular interface can be translated by the cytoskeleton and second messenger activities into gene expression and posttranscriptional modification of expressed proteins [61]. At this stage, it is evident that much of the natural environment surrounding osteoblasts and osteoclasts consist of structures with nano-scale topography. Following bionic principles, nanoscale surface architectures of grain sizes less than 100 nm attempt to recapitulate the physiological environment of growing bone. This results in enhanced osteoblasts spreading and filopodial extension. The detailed cellular response is sophisticated and varies according to the level of differentiation of the cell. While osteoblasts show enhanced differentiation on nanorough titanium, the effect of mesenchymal stem cells is controversy discussed. In a meta-analysis, Goldman et al. summarize the results of different investigators [62]. Especially the cellular Ca^2+^ influx, which is regulated by connexion 43 (Cx43), initiates similar cellular signals via gap junctions and is involved in osteoblast differentiation of MSCs [63].

**Phase V**: Local changes of the pH and accumulation of Ca and PO4 lead to biomineralization and osteointegration of the titanium implant. Here, osteoblasts and osteoclasts are mainly involved. At last biomechanical forces induce bone remodelling.

The detailed effects of micro- and nanoscale implant surface structures are not known and controversy discussed in the literature [64]. Most in vitro and in vivo studies suggest the minimal pore size required for bone tissue regeneration should be around 100 µm and pore sized below can lead to fibrous tissue formation or overgrowth with ossein. Especially pore sizes above 150 µm and functionally graded scaffolds favour bone formation and cell proliferation [65,66,67,68]. The influence of additive manufacturing to pore geometry, orientation and arrangement should also be considered in Ti implants. These procedures may affect mechanical strength as well as surface topography [69,70,71,72].

**Figure 2:**
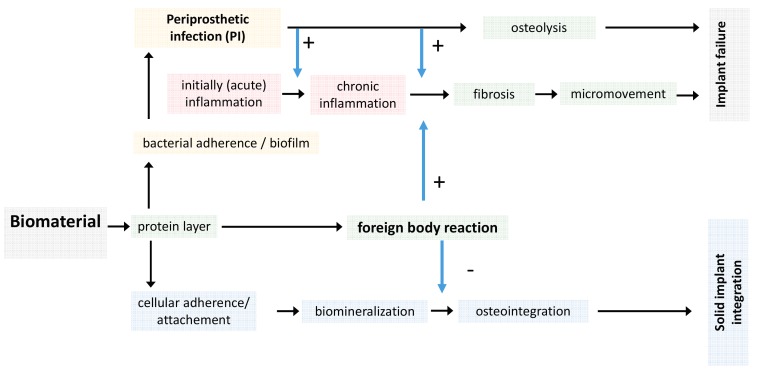
Local and systemic response after biomaterial application. The type of biomaterial and its surface structure dominantly influence osteointegration whereas an initial acute inflammatory reaction occurs independently and is based on the local surgical trauma.

Regardless of the type of biomaterial, an initial inflammation response occurs in vivo but subsides within few days postoperatively. In contrast to this, the foreign body reaction, the extent of fibrosis and the potency for cellular or bacterial adherence is dependent on the biomaterial properties (Figure 2).

The reaction of osteogenic bone cells to micro- and nanoscaled structured surfaces must first be elucidated in order to design the adequate coating for orthopaedic implants. In the following, the effects of microtopography, nanotopography, surface roughness, porosity and surface energy on osteogenic cell function and their consecutive influence on osseointegration are further described.

The bone cells surrounded by physiological osseous milieu and its connected topographical architecture consist of nanoscaled structures. Osteocytes, hydroxyapatite crystals and collagen fibrils, as predominant elements of the bone matrix, are in the range of 50–300 nm in length and 0.5–5 nm in width. Therefore, the design of nanoscaled implant surfaces is derived from its organic standard and simulates equal growing conditions for osteogenic cells [73].

**Figure 3:**
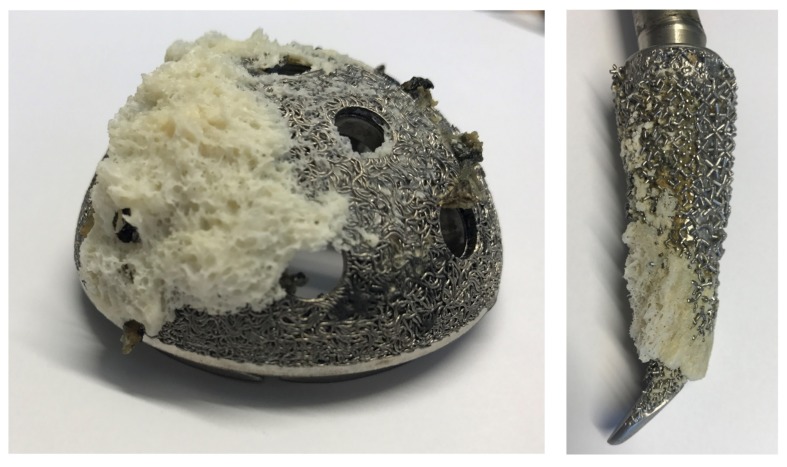
Macro- and microstrucured titanium implants show excellent biomechanical integration into bone in long-term. This implant-to-bone strength is higher than within the adjacent cancellous bone leading to intra-bone fracture during explantation. Here, de novo bone formation (contact osteogenesis) is based on micro-mechanical interdigitation of the surface with the material surface. It is believed that nanostructure titanium surfaces accelerate the initial phase of osteointegration leading to early full weight bearing even in poor bone quality. In contrast, the “bone-bonding phenomenon” is based on a chemical interaction of collagen, from the adjacent bone interdigitating with the chemically active surface of the implant [46,140].

However, even titanium implants are integrated close to the mineralized tissue. Some data suggest that titanium is separated by a very thin soft-tissue layer as a result of a weak foreign body reaction that prevents a direct contact its surface to the bone [7]. In well osteointegrated implants, these layers are missing (direct bone contact) [46] or have microscopic dimension leading to contact forces which are stronger than that of adjacent bone (Figure 3).

Nevertheless, the extent of this fibrosis is crucial for the clinical result: since encapsulation of titanium implants is not desirable for orthopaedic implants as it cannot withstand the physical stresses of daily activities, micromovements may result in chronic implant loosening or fracture. Therefore, there is enough room for further improvements of titanium surface properties to decrease inflammatory response, avoid foreign body reaction and promote cellular attachment and biomineralization. Nanostructures are one promising technology to reach these aims.

### 3.2. Titanium Nanostructures and Osteointegration

Cellular reactions (attachment, proliferation, differentiation, and protein synthesis) in vivo relay not only on the micrometre scale but also on the nanometre scale. Here, modifications of surface structures at the nanoscale level can improve the bioactivity and performance of titanium without changing its bioinert properties. Some of these effects are related to initial protein binding: shifting topography from the micro to the nanoscale increased surface energy leading to higher protein adsorption rates. In addition, nano-roughness alone influences the adhesion of osteoblasts and their spreading and proliferation (“rapid bone regeneration”) [74].

In vivo, microstructured surfaces provided a better implant surface–bone contact and an increased mechanical stability. The higher is the roughness, the higher are the local surface eletrostatic charge density and adhesion energy. Thus, it is possible to generate nanostructured titanium surfaces with superhydrophilic properties which can accelerate bone healing in early stages [75,76,77,78,79].

Meanwhile a large amount of diverse methods has been established to modify the surface structure of titanium implants. A typical example is the inorganic apatite coating, which can be controlled by plasmaspray techniques, electrodeposition or biomimetic precipitation of calcium phosphate (CaP immersion). During the last years, much more emphasis was put on the development of functionalized biomolecules onto titanium surfaces (e.g., collagen, fibronectin, protein fragments).

One example is the immobilization of ECM proteins such as fibronectin, collagen, osteopontin or laminin by plasma polymerized and crosslinked allylamine (pp-AA) [80] or the covalent binding of RGD-peptides [81]. In addition, polylactide (PLLA) coating and bound bone morphogenic protein (BMP) 2 seems to be one option for titanium surface structures with increased osteoinductive potential [82]. However, the critical point is the technical immobilization of these molecules to titanium and also the ability of the molecules to survive sterilization as well as surgical procedures.

Aiming for both optimization of osteointegration and prevention of bacterial adherence, a tremendous amount of research has been carried out into nanorough titanium implants. Especially defined nanostructures (nanotubes, nanorods, mesosponges, nanochannels, microcones, etc.) are promising candidates to improve implant properties [83]. To design these nanoscale structures electrochemical anodization, template method patterns, sol-gel processes or hydrothermal treatments are available. However, electrochemical anodization is one of the most established methods since it allows the formation of controlled surface structures such as nanotubes, pillar-like nanostructures, nanospikes and nanodots. The self-organization of Ti nanotubes in organic electrolytes depends strongly on its water content. Finally, this critical factor determinates whether self-ordered TiO_2_ nanopores or nanotubes were formed (“*pore wall splitting mechanism*”). Modifications in anodization conditions (voltage, anodization time, and concentration of chemicals) control the morphology such as diameter (15 nm–300 nm) or length of generated TiO_2_ tanotubes [84].

The growth rate and mechanical strength can be improved by high-voltage (HV) hard anodization (growth rate in HV: 1.2 μmh^−1^·V^−1^ vs. ~20 nmh^−1^·V^−1^ in LV regime) [85,86].

If the applied voltage exceeds the dielectric breakdown limit of the titanium oxide, the oxide will no longer be resistive to prevent further current flow and oxide growth. This leads to more gas evolution and sparking known as “Anodic Spark Deposition” (ASD), and “Micro-Arc Oxidation” (MAO) [87].

Among all electrolytes, the anodic oxide thickness is highest in H_2_SO_4_, but fluoride solutions seem to be more useful by producing biologically-inspired nano-tubular structures. Another major advantage of anodization is that nanotubes from almost all Ti alloys can be generated (e.g., Ti-6Al-7Nb, Ti-6Al-4V, Ti-6Al-4Zr). Interestingly nanotubes formed on Ti-6Al-4V by anodic oxidation have mainly been referred to as TiO_2_ nanotubes, while pure titanium oxide nanotubes are formed only on pure Ti [88,89,90]. Some impurities (e.g., V, Fe, F, C, N) derived from chemical etching solutions before anodizing. The detailed surface structure of nanotitanium processed by etching techniques is dependent on the etchant (e.g., acidic or basic Piranha solutions) and on the exposure time [40]. In contrast to anodization, where predominantly TiO_2_ nanotubes are created, the nanotubes formed on Ti-6Al-4V by thermal oxidation were referred to as Ti-Al-V-O nanotubes.

Besides morphological parameters, the degree of crystallinity changes the hydrophilic character of the nanotube. This has an impact on cell proliferation and osteointegration since nanostructured/hydrophilic surfaces show advantages [91,92,93,94,95]. Moreover, the formation of crystallic islands in the TiO_2_ nanotube structure accelerates growth in eukaryotic cells and reduces the ability of staphylococcal aggregate/biofilm formation. Nanotubes have a relevant size-effect on bone and other cells: Whereas diameters of 15–20 nm are optimal for increased cell adhesion, proliferation as well as for alkaline phosphatase (ALP) activity and bone matrix deposition [96,97,98,99,100], nanotobe diameters of approximately 100 nm led to programmed cell death (apoptosis). The hypothesis that hierarchical surfaces with smaller micro- and nanostructures mimicking nanoscale topography of the bone and thus promoting cell proliferation and osteoblast differentiation was confirmed by different investigators during the last decade [101,102,103,104].

Typical structures are bone apatite which forms 10–20 nm long and 2–3 nm wide plates, whereas type I collagen fibres are ~200 nm long and 2–3 nm thick [105,106].

This size-effect is valid for different substrate materials (e.g., TiO_2_ or ZrO_2_), for different crystallization states (amorphous and annealed) and is also unchanged in different fluoride contents in the tubes. It was confirmed on several cell types such as mesenchymal stem cells, haematopoietic stem cells, endothelial cells, osteoblasts and osteoclasts [84]. Nevertheless, alternative methods for anodization have been established to design and modify nanostructures (e.g., by ultrasonic fabrication of β-type Ti-Nb-Ta-Zr or by electrospinning of BaTiO_3_) [107,108].

The cause of the size effect is probably based on integrin clustering in the cell membrane leading to focal adhesion complexes. These have a diameter of about 10 nm which perfectly fit to nanotubular diameters of about 15 nm. In vivo, the cytotoxic effect of 100 nm is mitigated because larger nanotube dimensions show higher interaction energies, thus leading to higher protein adsorption which obviously influences cell behaviour [109]. Even in sharp nanorough titanium surfaces, the attraction between the negatively charged titanium and a negatively charged osteoblast is mediated by charged proteins with a distinctive quadrupolar internal charge distribution [75,76].

In addition, the cation-mediated attraction between extracellular matrix proteins (e.g., fibronectin) and the titanium surface is expected to be more efficient for a high surface charge density. This facilitated integrin-mediated osteoblast adhesion along the sharp convex edges or spikes of nanorough titanium surfaces where the magnitude of the negative surface charge density is the highest. In summary, Ti nanotubes with sharp edges and a diameter of approximately 15 nm should be the preferred surface for orthopaedic nanostructured implant. However, there are only few studies on whether in vitro effects of nanorough titanium can be transplanted to the in vivo situation.

Some other investigators described improved bone bonding strength and osteointegration in nanotubular surfaces compared with grit blasted surfaces [79,110]. These results were confirmed for TiO_2_ nanotubes (diameter: 37 ± 11 nm, thickness: 160 nm thick) by pull-out experiments in rabbits [111,112]. Moreover, the osteointegrative nature of Ti nanotubes can be improved by further modifications such as Si-doped TiO_2_ (TiO_2_-NT) [113] or Ti-24Nb-4Zr-7.9Sn [114].

In summary, most in vivo data suggest that especially the initial bone formation is enhanced in nanorough titanium surfaces [111]. This statement has a relevant clinical impact because patients with poor bone quality (e.g., elderly people) might benefit from earlier full weight bearing after cementless fixed total joint.

One further option for changing surface properties of titanium is the implantation of bioactive ions such as Ca, Zn, H and Sr. Hegedus et al. found improved cell viability for Ti with implanted Ca or dual Ca + Si ion compared to pristine Ti substrate [115]. This corresponds to the findings of Wang et al. who showed advantages in osteointegration for Ca and Ta entangled porous titanium (EPT) by plasma immersion ion implantation and deposition [116]. It is believed that Ta–OH groups can facilitate the adsorption of calcium and phosphate ions, thus enhancing osteoblasts adhesion. Gold (Au) is also qualified to optimize Ti surfaces aiming for osteointegration as shown for gold ion implantation, thin Au layer deposition and thermal annealing [117,118,119].

More complex for getting official approval as a medical device is the covalent immobilization of biomolecules e.g., BMP-2 onto nanostructured Ti surfaces. Experimental data have shown that this can be a useful tool to control differentiation of mesenchymal stem cells (MSCs). Typical examples are:

- immobilization of epidermal growth factor (EGF) on 100 nm diameter TiO_2_ nanotubes (prevention of apoptosis, promotion of attachment and proliferation) [120];
- immobilization of bone BMP-2 onto TiO nanotubes:
  ○ higher osteocalcin and osteopontin levels at 30 nm diameter [121]; and
  ○ enhanced osteogenic differentiation 15 nm diameter, chondrogenic differentiation on 100 nm [98]; and
- vascular endothelial growth factor (VEGF) [122].

This functionalization could be performed directly, but usually is realized via self-assembled monolayers (SAMs), antibodies or peptides on the surface of nanostructures [123]. The concept of bio-functionalization of metal surface structures is not new but still an increasing area of research [124].

Especially the pharmacokinetics of Ti-bound BMPs is hard to control in vivo. This is relevant, since extensive concentration of local released BMP can induce osteolysis [125,126,127,128]. Aiming for steady release over a longer period, Ti surfaces coated with graphene oxide through layer-by-layer assembly of positively and negatively charged grapheme oxide sheets guarantees high load dosages of BMP-2. This and biodegradable-loaded BMP-2 carriers have shown promising results in vitro and in animal experiments [129,130,131,132,133].

However, it has been taken into account that the secondary structure of rhBMP-2 can change significantly dependent on the modified surface (e.g., decreased percentage of α-helix structure in acid alkali-treated Ti). These conformational changes will also influence the BMP-2 activity [134]. Another strategy is to improve the osteoinductive properties of Ti implants to activate the TGF-β/BMP and non-canonicalWNT/Ca^2+^ (WNT5A, FZD6) pathways directly by modified surface structures as shown by Chakravorty et al. [135]. Especially in vitro results require highly standardized animal models to receive reliable data [136].

The latest development of nanostructured titanium aims at osteopromotion and prevention of bacterial adhesion. One example is to design hierarchical architectures by a “layer-by-layer” (LbL) concept. Here, chitosan coated BSA nanoparticles (CBSA NPs) and oxidized alginate (OSA) showed features of nanostructures on titanium. The functional groups provide active sites for BMP-2-immobilization and promote bone formation. Furthermore, it was shown that the antibiotic vancomycin binds on the OSA. Therefore, these assembling nanoparticles might prevent infection during the bone healing process [137].

However, the application of local antibiotics to nanostructured titanium for clinical application seems to be critical regarding pharmacological side effects including bacterial resistance, limitations in implant sterilization and official approval.

More promising is the combination of a titanium surface structure with other agents such as HA or iodine as described by Tsuchiya et al. [138,139]. The authors report good clinical results (osteointegration and infection treatment) in 222 patients using an iodine filled nanopore titanium surface with an oxide film of 5 and 10 µm (>50,000 pores/mm^2^).

The following section summarizes further anti-microbiological effects of nanotitanium structured surfaces and clinical strategies to treat infections.

## 4. Periprosthetic Infections and Biofilm

The demographic change in the Western world and prolonged life expectance have increased the rate of total joint revision operations dramatically [141]. Implant failures due to PI or surgical site infections (SSI) are the third most common cause in total hip arthroplasty (15%) and the most common cause in total knee arthroplasty (25%) [142,143].

The consequences of PI are an imprinting and traumatic experience for the patient. Radical debridement of soft tissue and bone during revision surgery leads to progressive bone loss and therefore loss of biomechanical stability. The revision operation increases the complication rate compared to primary implantation significantly [144]. Furthermore, the cases of infections with multiresistant bacteria are increasing and the pharmacological options are stretched to its limit. In addition to the physical and psychological impairment of the patient, there is an economic burden on the health system caused by PI revision surgery that cannot be neglected.

The pathogenesis of PI or septic loosening is due to intraoperative contamination of the implant or the periprosthetic interface, which may become apparent in an early-onset acute local putrid infection, a Systemic Inflammatory Response Syndrome (SIRS) or in a “low-grade infection”. In addition to the direct contamination, a secondary infection of the prosthetic joint by bacteraemia e.g., after soft tissue infection, pneumonia or tooth extraction is also possible [145].

The initial contamination of the biomaterials surface is followed by the development of a biofilm on the colonized surfaces. Biofilms are “aggregates of microorganisms in which cells are frequently embedded in a self-produced matrix of extracellular polymeric substances that are adherent to each other and/or a surface”. Typically, biofilms comprise many species and are complex systems with high cell densities [146]. This biofilm is critically influencing the implants function itself and initiates an periprosthetic inflammation [147].

Innovative solutions for biofilm absorbing or antibacterial Ti surfaces were achieved by permanent binding of antibiotics [148], antimicrobial peptides [149], antibacterial nanoparticles [150] and nanosilver or sputtered silver compounds [151]. Indeed, more effective in preventing PIs are topographical changes of the biomaterials surfaces on a micro- or nanoscaled level to prevent the growing risk of antimicrobial resistance without using any additional chemicals or antibiotics. It is generally accepted that nanoscaled Ti with 20–80 nm surface features decreases the bacterial adhesion significantly [152].

Mechanisms for these inherent antimicrobial properties are part of current research. It is hypothesised that defined nanotopographies are able to disrupt bacterial cell membranes and lead to a decreased bacterial viability. An effective microbial lysis is achieved by creating topographies which generate an adequate local stress across the microbial cell membrane. Dickson et al. defined the ideal space in between nanopillars in the range of 130–380 nm [153]. In addition, Gorth et al. pointed out that sharp edges on grains of Si_3_N_4_ surfaces are also capable of bacterial lysis. Thus, the topographical bactericidal effect is primarily dominated by its surface nanotopography and not only by its surface biochemistry [154]. Sjöström et al. underlined this hypothesis by using thermal oxidation to manufacture a range of nanospikes with a diameter of approximately 20 nm on the surfaces of Ti alloy beads. The nanospikes in the 20 nm range decrease the viability of *Escherichia coli* on the surface by 40% in comparison with polished Ti in vitro [155].

Mathew et al. showed that greater nanoscale surface roughness led to significantly decreased bacterial colonisation. In detail, implants coated with electrophoretic-deposited hydroxyapatite inhibited the growth of *Staphylococcus aureus* more effectively compared to less nanoscaled Ti surfaces [156].

Recently, a new potential antibacterial phenomenon based on solely nanotopographical properties has been found in locus-like insects. The wings of Clanger cicada (*Psaltoda claripennis*) are covered by hexagonal arrays of nanopillars which are able to eliminate Gram-negative bacteria by its physical surface structure consistently [157]. The antimicrobial effect is again due to rupture of the bacterial cell membrane and independent from its biochemistry. The nanopillars’ height and spacing is about 200 nm and 100 nm in diameter at its base and covered by a self-cleaning wax layer, which leads to a super hydrophobic surface [158]. The “cicada wing effect” can be translated into titanium biomaterials by imitating the nanocolumnar structures using glancing angle sputter deposition (GLAD). In conclusion, a selective antimicrobial effect, using the GLAD technique, against Gram-negative bacteria, e.g., *Escherichia coli*, could be shown, whereas Gram-positive bacteria, e.g., *Staphylococcus aureus*, were not affected [159]. Table 3 summarized different Ti surface structures and its effects on prokaryotes.

Another example how surface topography can affect bacterial growth are “rose stem-like constructs”: The combination of anisotropic branched-shaped zinc oxide (ZnO) nanoparticles with fibrous scaffolds such as polycaprolactone (PCL) fabricated by electrospinning lead to protrusions mimicking the architecture of a rose stem. The branched nanoparticles (spikes length: 1–5 μm; diameter: 50–200 nm) induced heterogeneous crystallization of the polymeric matrix. This three-dimensional composite enhances the mechanical strain and strength and offers excellent antibacterial activity, while supporting the growth of eukaryote cells [170]. Bounded to Ti surfaces these structures might improve osteocondutivity and inhibit proliferation of prokaryotes. In addition, at the micoporous level prototypes of such hierarchical microspikes have shown excellent osteopromoting properties in vitro and in vivo [171]. However, if the bactericidal activity of the micro- and nanoscale structure itself is not potent enough, other mechanisms are required to accelerate the antimicrobial properties of Ti surfaces. Embedding of additional ions such as Ag, Cu, Arg or Ga is one promising option. The major challenge is to create surfaces that are harmless to eukaryotic cells but show antimicrobial activity.

The bactericidal effect of silver is based on interactions of Ag^+^ with bacterial membrane constituents. This causes structural changes, interrupts transmembrane electron transfer, oxidizes bacterial components and may at least induce bacterial death. In addition, Ag^+^ shows also cytotoxic effects such as mitochondrial dysfunction, disturbed membrane integrity, induction of reactive oxygen species, and interrupted adenosine triphosphate synthesis resulting in DNA lesions. The cytotoxic effects of Ag^+^ depend on the instability of Ag-based bactericides, such as the high mobility of Ag nanoparticles (NPs) or Ag-containing calcium titanate. This can be avoided by silver plasma immersion ion implantation (Ag-PIII) with atomic-scale heating (ASH). The three-dimensional, hierarchical structure of sand-blasted, large grit, and acid-etched (SLA) Ti surfaces showed optimal preconditions for silver plasma immersion ion implantation. These techniques showed not only a sufficient defence against multiple cycles of bacterial attacks, independently from silver release, but they are also nontoxic to eukaryotic cells such as bone marrow mesenchymal stem cells [172,173,174,175].

Another strategy to combine antimicrobial and osteoprotective properties is the incorporation of gallium (Ga) ions in Ti surfaces. In contrast to Ag, Ga showed higher cytocompatibility since it can substitute Fe in many biological systems (same charge 3+, similarities in ionic radius and electronic configuration) and inhibits bone resorption. In bacteria Ga^3+^ acts also as a “Trojan horse” competiting with Fe^3+^ for binding to siderophores, thus interrupting crucial Fe-dependent metabolic pathways. One method embedding Ga into Ti surfaces is based on chemical and heating treatments resulting in a Ga-containing calcium titanate (Ga–CT) or gallium titanate (GT) surface. It was demonstrated that Ga–CT and GT interfaces exhibited very high antibacterial activity against multidrug resistant *Acinetobacter baumannii* (MRAB12) and inhibits bone resorption [176,177,178,179]. However, there are no clinical trials with the “Two-in-One Biointerface” available so far. Other authors are embedding Ti nanoparticles to improve the antimicrobial effects of polymers, which have a potential for orthopaedic application. Typical examples are carbon-fibre-reinforced polyetheretherketone (CFRPEEK) [180] or poly lactic acid (PLA) [181].

**Figure 4:**
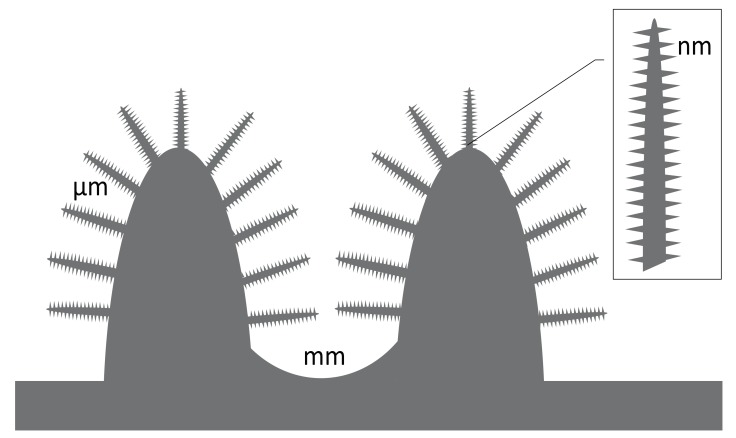
The hierarchical structure of a typical implant surface includes different levels of roughness. The morphology of each dimension is dependent from the material composition, the manufacturing process and post processed changes. Defined rose stock, brush-like or multi-spike topographies showed osteoconductive and bactericidal properties. It is hypothesized that these bioinspired nanospikes designs will deform or rupture the bacterial cell wall and lead to death (physical killing mechanism) [184].

Inspired by bacteriophages that use nanostructured proteins to invade bacteria, other investigators study the antibiotic role of nanostructures. Mimicking the two basic structural motifs of bacteriophages, spherical and rod-like polymer molecular brushes (PMBs) have been designed. However, the impact of these techniques for Ti implants is unclear in future [182,183]. However, for the practical performances the two-dimensional surface structures such as nano-grits, nano-tube, or nano-ripple are critical (Figure 4 and Table 4).

However, for practical performance in orthopaedic surgery, the narrow valleys or slots of nanomaterials might be filled up with adsorbed proteins and other molecules within few minutes. Therefore, it also seems to be questionable if the antimicrobial effects of the nanostructures (e.g., nano-grits, nano-tube, or nano-ripple) will have a relevant and persisting effect on prokaryotes. Besides, appropriate mechanical strength of nanostructures is a requirement for clinical application. For some materials infinite element analysis is available to predict the mechanical behaviour [188]. Especially in press fit implants, these structures have to withstand relevant shear forces during application and also load bearing.

## 5. Outlook and Recent Trends in Nanostructured Ti

From our point of view, the future in nano-based orthopaedic Ti implants will stronger focus on NPs composites such as PLA electrospun fiber-TiO_2_ nanoparticle [189] and also on custom-made scaffolds fabricated by additive manufacturing (AM) methods. Besides composites, also coating techniques such as sialinisation-mediated binding have a strong potential to improve cellular binding and biomineralization as well as infection prophylaxis. In this context we doubt that antibiotics are promising coating candidates for Ti implants [190]. Besides regulatory burdens, clinical effects might be complicated by molecule instabilities (sterilization, storage, and shelf life), biomechanical forces (in vivo application), and local bacterial resistances. Nevertheless, from the evolution point of view there is also evidence that biological systems respond to novel materials and develop ion escape strategies. Some investigators emphasize that their surface modification decrease the release of cytotoxic metal ions, but there are no data available yet on the possible role of metal ions in the development of bacterial resistance in sub-minimal inhibitory concentrations (MIC).

Many bacteria express metal-sensing transcriptional regulators allowing them to adapt to their environment rather quickly. Within minutes to hours, bacteria are able to escape toxic metals such as Ag^+^, Cd^2+^, Hg^2+^, Ni^2+^, etc. (physiological acclimation). These species and strain dependent strategies include:

- energy dependent efflux of toxic metals e.g., enzymatic transformations (oxidation, reduction, methylation, and demethylation);
- the expression of metal-binding proteins (metallothionein, SmtA, chaperone CopZ, SilE);
- inhibition of ions to enter the cell (downregulated expression of membrane transport proteins); and
- persisting phenotypes with slower growth or ceasing cell division [191].

In contrast to these initial physiological responses to toxins, which is described for *E. coli* and Ag^+^ [192], there is always genetic variation among the microbes leading to natural selection. The result is an evolutionary adaption which occurs rapidly.

In contrast to today’s implants, future Ti surfaces will have more controlled surface design with less variation on the macro-, micro- and nanoscale. For many years, the pharmaceutical industry designed drugs on a molecular level. Future medical devices such as Ti implants might follow these developments to the benefit of orthopaedic patients. Rapid Prototyping (RP) Technologies (e.g., 3D fibre deposition), electron beam melting (EBM), selective laser melting (SLM) or 3D printing can fabricate porous Ti scaffolds with fully interconnected porous network and highly controllable porosity and pore size [193,194,195,196,197,198,199].

Understanding the molecular principles and interactions at the tissue-surface interface at different dimensional levels (porosity gradient) might help closing the gap between promising new designed Ti structures of the research stage (“valley of dreams”) and the so-called “valley of death” of further translational and preclinical stages of implants. One example is to control cell performance to a certain extent by varying pore sizes [69,200]. Smart, multicomponent surface systems capable of delivering both local osteoinductive and bactericidal stimuli will make the breakthrough and revolutionize orthopaedic implants.

## Figures and Tables

**Table 1:** Different metal alloys and their density and Young’s modulus in comparison to bone [2,3,4,5,6].

Material	Density (Mg/m^3^)	Young’s Modulus, E (GPa)
Cp-Ti grade II	4.2	100–110
Ti-6Al-4V	4.5	100–130
Ti-6Al-7Nb	4.52	110–130
Surgical CrNiMo-Steel 316L	7.8	195–210
CoCrMo alloys	8.5	210–230
Cortical bone	1.19–1.85	18.6–20.7

**Table 2:** Commonly applied methods to modify titanium surface structures into the nanoscale in the orthopaedic field [7,8,9,10,11,12,13,14,15,16,17,18,19,20,21,22,23,24].

	Technique	Modified Layer	Objective
**Mechanical**	Grinding Polishing Machining Blasting	Rough or smooth surface formed by the subtraction process	Produce specific surface topographies; Clean and roughen surface; Improve adhesion in bonding
	Attrition	To fabricate nanophase surface layers on Ti of commercial purity which improve the tensile properties and surface hardness of Ti	Produce materials with nanometre size grains (1–100 nm); To produce rough morphology and higher hydrophilicity
**Chemical**	Acidic treatment Alkaline treatment Hydrogen peroxide treatment	<10 nm of surface oxide layer ~1 μm of sodium titanate gel ~5 nm of dense inner oxide and porous outer layer	Remove oxide scales and contamination. Improve biocompatibility, bioactivity or bone conductivity. Improve biocompatibility, bioactivity or bone conductivity
	Sol-gel	~10 μm of thin film, such as calcium phosphate, TiO_2_ and silica	Improve biocompatibility, bioactivity or bone conductivity
	CVD	~1 μm of TiN, TiC, TiCN, diamond and diamond-like carbon thin film	Improve wear resistance, corrosion resistance and blood compatibility
	Anodic oxidation	~10 nm–40 μm of TiO_2_ layer, adsorption and incorporation of electrolyte anions	Produce specific surface topographies; improve corrosion resistance; improve biocompatibility, bioactivity or bone conductivity
	Biochemical methods	Coating deposition; modification through silanized Ti, photochemistry, self-assembled monolayers, protein-resistance, etc.	Induce specific cell and tissue response by means of surface immobilized peptides, proteins, or growth factors
**Physical**	Thermal spray ✓ flame spray ✓ plasma spray ✓ high velocity oxy-fuel spray ✓ others	~30 to ~200 μm of coatings, such as titanium, HA, calcium silicate, Al_2_O_3_, ZrO_2_, TiO_2_	Improve wear resistance, corrosion resistance and biological properties (osteoblast adhesion)
	Physical vapour deposition ✓ Evaporation ✓ Ion plating ✓ Sputtering	~1 μm of TiN, TiC, TiCN, diamond and diamond-like carbon thin film Hydroxyapatite coating by sputtering	Improve wear resistance, corrosion resistance and blood compatibility.
	Ion implantation and deposition	~10 nm of surface modified layer and/or um of thin film	Modify surface composition; improve wear, corrosion resistance, and biocompatibility
	Glow discharge plasma treatment	~1 nm to ~100 nm of surface modified layer	Cleaning, sterilizing or oxidizing the surface; surface nitridation; removal of the native oxide layer

**Table 3:** Nanostructured antibacterial titanium surfaces (Ag: silver, Zn: zinc, Fe: iron, TiN: titanium nitride, MRSA: Methicillin-resistant *Staphyloccus aureus*) [160].

	Method	Effect	
**Coating**	Ag-nanoparticle modified Ti by silanization	Decreased viabilitiy and adhesion of *Escherichia coli* and *Staphylococcus aureus* in vitro	[161]
	Poly(quaternary ammonium)-modified gold and TiO_2_ nanoparticles	Decreased viability of *Escherichia coli* (5 logs in 10 min) in vitro	[162]
**Surface structure**	Nanophased ZnO and TiO_2_	Decreased adhesion of *Staphylococcus epidermidis* in vitro	[163]
	Nanoscaled TiN/Ag multi-layered films on Fe (modulation period 7.5 nm)	Bactericidal in *Escherichia coli* in vitro	[164]
	Electrospun TiO_2_ nanorods by sol-gel electrospinning technique	Disruption of cell membrane in *Escherichia coli*, *Salmonella* Typhimurium, *Klebsiella pneumoniae*, *Staphylococcus aureus* in vitro	[165]
	Zn-doped Ti nanofibers	Disruption of cell membrane in *Escherichia coli*, *Staphylococcus aureus* in vitro	[166]
	Nanopillars/Nanotubes on Ti “cicada wing effect”	Disruption of cell membrane in *Escherichia coli* in vitro	[159]
	Nanostructured Zn-incorporated TiO_2_	Decreased growth of *Escherichia coli* and *Staphylococcus aureus* in vitro	[167]
	Ag/TiO_2_ nanocomposite powder by one-pot sol-gel technique (Np < 2 nm)	Complete growth inhibition of *Escherichia coli* in vitro	[168]
	Nanostructured sodium silver titanate (nanotube) thin films	Antibacterial against MRSA in vitro	[169]

**Table 4:** Natural nanostructures and bio-inspired Ti structures modified from [155,184,185,186,187]. In contrast to hydrophilic surfaces hydrophobic surfaces prohibit bacterial growth as the bacteria cannot stick to the surface. Defined nanostructured surfaces can stretch and rupture the relatively thin bacterial cell wall. This rapid morphological change of the adhered bacteria induce its death within a few minutes (approximately 3–5 min, “contact killing mechanism”). However, the peptidoglycan layer of the cell wall of Gram-positive (G+) bacteria is 4–5 times thicker than that of Gram-negative (G−). Here, defined surface textures of the nanostructure are required to enfold its bacteriostatic effects which are based on reduced adhesion forces. A special challenge and exception are mycobacteria. In contrast to many other bacteria their cell wall is thicker, hydrophobic, and rich in mycolic acids. It is believed that, for this reason, there has not been any study on the bactericidal efficiency of nanostructured surfaces. HY: hydrophobic, HP: hydrophilic, CA: contact angle.

Surface	Surface Feature	Method	Wettability (CA)	Bactericidal and Fungicidal Efficacy
*Cicada wing*	Nanoneedles, height 200 nm, diameter 60 nm size at the top, 100 nm at the base of the pillar, and spacing 170 nm	natural	HY [159°]	Lethal to *P. aeruginosa* (G−)
*Gecko skin*	Hair (spinules) like structures with sub-micron spacing and a tip radius of curvature <20 nm	natural	HY [151°–155°]	Lethal to *Porphyromonas gingivalis* (G−)
*Dragon fly wing*	Nanograss, diameter 50–70 nm, height 240 nm	natural	HY [153°]	Lethal to *P. aeruginosa* (G−), *S. aureus* (G+), *B. subtilis* (G+)
*Periodical cicada*	Hemispherical nano features with height 83.5 nm, diameter 167 nm, pitch 252 nm	natural	HP [80.1°]	Caused cell wall rupturing of *Saccharomyces cerevisiae*
*Annual DD cicada*	Spherical nanocones with height 183 nm, base diameter 104 nm, cap diameter 104 nm, pitch 175 nm	natural	HY [132°]	Caused cell wall rupturing of *Saccharomyces cerevisiae*
*Sanddragon dragonfly*	High-aspect ratio spherical capped nanocylinders with height 241 nm, diameter 53 nm, pitch 123 nm	natural	HY [119°]	Caused cell wall rupturing of *Saccharomyces cerevisiae*
*Megapomponia intermedia*	Nanopillars with height 241 nm, diameter 156 nm, pitch 165 nm	natural	HY [135.5°]	Bactericidal against *Pseudomonas fluorescens* (G−)
*Cryptotympana aguila*	Nanopillars with height 182 nm, diameter 159 nm, pitch 187 nm	natural	HY [113.2°]	Bactericidal against G− *P. fluorescens*
*Ayuthia spectabile*	Nanopillars with height 182 nm, diameter 207 nm, pitch 251 nm	natural	HY [95.65°]	Bactericidal against *P. fluorescens* (but more than *Megapomponia intermedia* and *Cryptotympana aguila)*
Titania nanowire arrays	Nanowires, brush type: Diameter 100 nm	Hydrothermal	-	Effective in killing motile bacteria (*P. aeruginosa*, *Escherichia coli* (G−), *B. subtilis*), less lethal against non-motile bacteria (*S. aureus*, *Enterococcus faecalis* (G+), *K. pneumoniae (G−)*)
Titania nanowire arrays	Nanowires, niche type: Diameter 10–15 μm	Hydrothermal	-	Effective in killing motile bacteria (*P. aeruginosa*, *Escherichia coli* and *B. subtilis*), less lethal against non-motile bacteria (*S. aureus*, *Enterococcus faecalis*, and *Klebsiella pneumoniae*)
Ti nanopatterned arrays	Nanopatterned arrays, average diameter 40.3 nm	Hydrothermal etching	*HP [73°]*	Effective in killing *P. aeruginosa*, less lethal against *S. aureus*
Ti alloy nanospike surface	Nanospikes, average diameter 10 nm, spacing 2 μm, height 2 μm	Anodization	-	Lethal to *S. aureus*
Ti alloy anospike surface	Nanospikes, average diameter 20 nm	Thermal oxidation	-	Lethal to *E. coli*

## Abbreviations

AM	additive manufacturing
ASH	atomic-scale heating
Ag	argentum (silver)
Al	aluminium
ALP	alkaline phosphatase
*B*.	Bacillus
Ba	barium
BMP	bone morphogenic protein
BSA	bovine serum albumin anoparticles
C	carbon
Ca	calcium
CA	contact angle
CFRPEEK	carbon-fiber-reinforced polyetheretherketone
Cd	cadmium
copZ	copper transport protein, metallochaperone, Crk-associated substrate
cp	commercial pure
Cr	chromium
*E*.	*Escherichia*
EFG	epidermal growth factor
EPT	entangled porous titanium
F	flour
FAK	fokal adhesion kinase
FN	fibronectin
ECM	extracellular matrix
G−	gram negative
G+	gram positive
Ga	gallium
Ga–CT	Ga-containing calcium titanate
GT	gallium titanate
ERK	extracellular signal-regulated kinase
Fe	ferrum (iron)
GLAD	glancing angle sputter deposition
H	hydrogen
Hg	hydrargyrum (mercury)
HA	hydroxyapatite
Hf	hafnium
HP	hydrophilic
HV	high voltage
HY	hydrophobic
*K*.	*Klebsiella*
LV	low voltage
Mo	molybdenum
MEK	MAPK/Erk kinase
MIC	minimal inhibitory concentration
MLC	myosin light chain
N	nitrogen
Nb	niobium
NMATF	nuclear matrix architectural transcription factors
NPs	nanoparticles
O	oxygen
OSA	oxidized alginate
P	phosphorus/phosphate
*P*.	*Pseudomonas*
PCL	polycaprolactone
PI	periprosthetic infection
PLLA	polylactide
PMB	polymer molecular brushes
RANKL	receptor activator of nuclear factor kappa-B ligand
Rho	Ras (Rat sarcom) homologue
RGD	Arginylglycylaspartic acid (tripeptide composed of l-arginine, glycine, and l-aspartic)
ROCK	Rho-associated protein kinase
*S*.	*Staphylococcus*
SAMs	self-assembled monolayers
SLA	sand-blasted, large grit, and acid-etched
Si	silicium
SilE	periplasmic Ag(I)-binding protein expressed by a sensor/responder two-component transcriptional regulatory system
SIRS	systemic inflammatory response syndrome
Sr	strontium
Ta	tantalum
Ti	titanium
V	vanadium, vascular endothelial growth factor (VEGF)
Zn	zinc
Zr	zirkonium
